# Strong-field photoelectron holography in the subcycle limit

**DOI:** 10.1038/s41377-024-01457-7

**Published:** 2024-05-08

**Authors:** Tsendsuren Khurelbaatar, Jaewuk Heo, ShaoGang Yu, XuanYang Lai, XiaoJun Liu, Dong Eon Kim

**Affiliations:** 1 Center for Attosecond Science and Technology, Department of Physics, Pohang University of Science and Technology, Pohang, Gyeongbuk 37673 Korea; 2 Max Planck POSTECH/KOREA Research Initiative, Pohang, Gyeongbuk 37673 Korea; 3 State Key Laboratory of Magnetic Resonance and Atomic and Molecular Physics, Wuhan Institute of Physics and Mathematics, Innovation Academy for Precision Measurement Science and Technology, Chinese Academy of Sciences, 430071 Wuhan, China; 4 Wuhan Institute of Quantum Technology, 430206 Wuhan, China

**Keywords:** Nonlinear optics, High-harmonic generation

## Abstract

Strong-field photoelectron holography is promising for the study of electron dynamics and structure in atoms and molecules, with superior spatiotemporal resolution compared to conventional electron and X-ray diffractometry. However, the application of strong-field photoelectron holography has been hindered by inter-cycle interference from multicycle fields. Here, we address this challenge by employing a near-single-cycle field to suppress the inter-cycle interference. We observed and separated two distinct holographic patterns for the first time. Our measurements allow us not only to identify the Gouy phase effect on electron wavepackets and holographic patterns but also to correctly extract the internuclear separation of the target molecule from the holographic pattern. Our work leads to a leap jump from theory to application in the field of strong-field photoelectron holography-based ultrafast imaging of molecular structures.

## Abstract (web summary)

Fundamental electron-holograms in the single-cycle limit are clearly unveiled to identify electron Gouy-phase and extract the internuclear separation.

## Graphical abstract

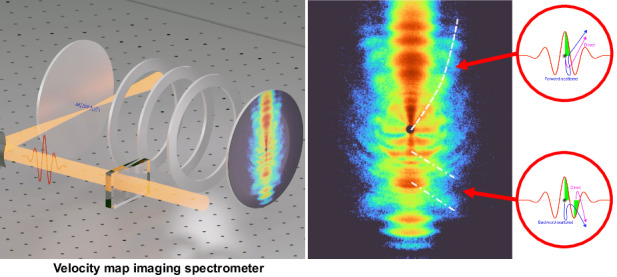

## Introduction

The understanding and visualizing transient molecular structures have been pursued in the field of ultrafast science. Conventional electron and X-ray diffraction methods have been employed for high-resolution imaging of molecules with angstrom-level precision; however, their temporal resolution has remained limited to femtosecond timescales, primarily capturing nuclear dynamics. Recent advancements in laser technology have opened new avenues for tabletop laser-based imaging methods such as ultrafast electron diffraction, laser-induced electron diffraction, and strong-field photoelectron holography (SFPH)^1–6^. Among them, the SFPH has gained widespread attention owing to its remarkable capability to provide rich information about the target structure and underlying dynamic processes, offering superior sub-angstrom spatial and attosecond temporal resolutions^7–12^.

In the past, the study of the SFPH method has allowed us to identify intriguing holographic patterns such as “spider-leg-like” and “fishbone-like” holograms, among others^13–16^. These patterns harbor invaluable information about molecular arrangements, prompting researchers to explore methods to accurately reconstruct molecular structures from holograms. However, the extraction of structural information with SFPH is far from straightforward because of the intricate interplay between interference phenomena, which often obscure and distort the desired information. A primary obstacle responsible for these challenges lies in the inter-cycle interference of electron wavepackets within the strong multicycle laser fields^17^, which intertwine with holographic patterns of interest and significantly impact the quality of the hologram.

To alleviate the limitations encountered in the multicycle regime, the use of sophisticated experimental methods, such as two-color laser fields^18–21^, differential holographic measurement^22^, and intricate data analysis methods^23,24^, has been proposed; however, direct observation of target structure-relevant holographic interference patterns remains a challenge owing to the persistent influence of inter-cycle interference effects. In light of the challenges posed by inter-cycle interference, the use of single-cycle laser pulses emerges as a viable alternative for SFPH studies^25–27^. Unlike multicycle laser fields, single-cycle pulses have shorter durations and well-defined oscillation periods. These characteristics allow the isolation of individual laser-matter interactions within a single optical cycle, thereby reducing the complexity introduced by inter-cycle interference effects.

In this work, using a carrier-envelope-phase (CEP)-stabilized, near-single-cycle laser pulse, we get rid of the inter-cycle interference effect, allowing two distinct holographic patterns, spider-leg-like and fishbone-like, to be observed and separated in photoelectron momentum space for the first time. The experimental findings were qualitatively well reproduced by time-dependent Schrödinger equation (TDSE) simulations, and the underlying physics for the appearance of the two photoelectron holographic (PH) patterns in the near-single-cycle laser pulse revealed by developing a theoretical method. By using the two observed PH patterns, a Gouy phase effect on the ionized electron focused by Coulomb attraction was found. Moreover, we showed that the fishbone-like PH pattern is sensitive to the molecular structure, and the fringe positions can be used to fit the internuclear separation of the molecule. Therefore, based on the observation of inter-cycle interference-free isolated PH patterns, our method can further be used for the extraction of target structure information at high spatiotemporal resolutions.

## Results

### Direct observation and TDSE simulation of fishbone- and spider-leg-like PH patterns

**Fig. 1:**
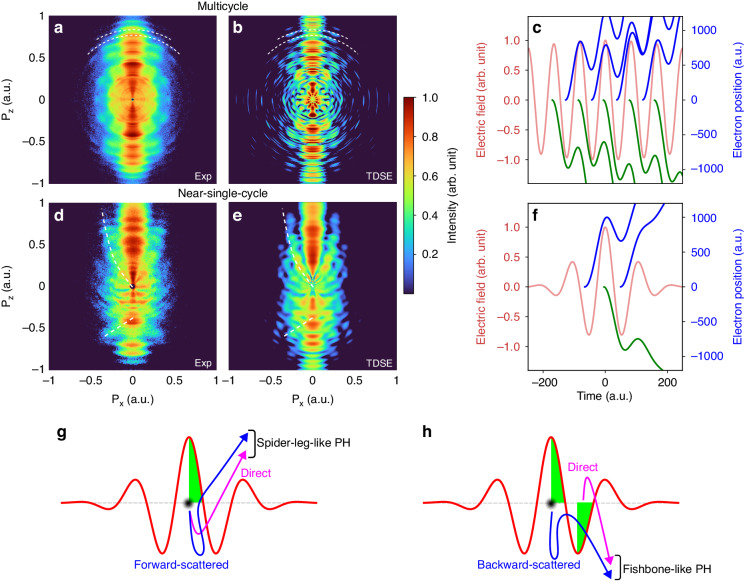
Observation of spider-leg- and fishbone-like PH patterns. Measured (**a**, **d**) and TDSE-simulated (**b**, **e**) PH patterns from nitrogen molecules ionized with (**a**, **b**) multicycle and (**d**, **e**) near-single-cycle laser pulses. For a proper comparison with experimental data, the TDSE simulations have been done for laser intensities corresponding to the spatial profile of a Gaussian laser focus, and then have been integrated over the spatial profile of laser focus. **c**, **f** Illustrate classical electron trajectories (blue and green) that are ionized every half cycle of the electric field (red). **c** A portion of a 25-fs Gaussian pulse is considered to display classical electron trajectories. For simplicity, only a single ionization event per half cycle is considered, and direct electron trajectories are shown in both cases. **g**, **h** Depict a simplified scheme for the underlying mechanisms contributing to the corresponding observed PH pattern

A series of experiments were carried out using a high-resolution velocity map imaging (VMI) spectrometer^28^ with a CEP-stabilized near-single-cycle laser (see Supplementary information). Figure 1a, d shows the measured (Abel inverted) PH patterns from N_2_ molecules for different pulse durations. Figure 1a was obtained from a multicycle laser pulse (25 fs, 800 nm), and Fig. 1d from a near-single-cycle laser pulse (3.3 fs, 723 nm). Figure 1b, e illustrates the TDSE simulation results. For the comparison with the experimental data, the simulation takes into account the focal-volume averaging of the laser beam for the respective pulse durations (see Supplementary information). Here, the pulse durations are determined at the intensity full width at half maximum (FWHM). Figure 1c, f illustrates the effects of cycle number for the SFPH experiment, and Fig. 1g, h schematically displays the origin of the observed interference pattern.

The major difference in the PH patterns between multicycle and near-single-cycle pulses is the above-threshold ionization (ATI) peaks, which arise due to inter-cycle interference^17,29^. The origin of this interference pattern is depicted in Fig. 1c, considering a portion of a 25-fs Gaussian pulse at 800 nm. As shown in Fig. 1c, electrons can be ionized every half cycle of the laser field, and electron pairs that are ionized at least one full cycle apart and emitted in the same direction are able to coherently interfere and produce interference patterns (blue and green trajectories). On the other hand, electron pairs produced within a single optical cycle are also able to coherently interfere when rescattered. This is referred to as intra-cycle interference (i.e., a pair of blue and green trajectories). Therefore, the measured PH pattern in Fig. 1a shows rich symmetric interference patterns originating from both inter- and intra-cycle dynamics, such as ATI peaks (white dashed arcs) and spider-leg-like PH patterns. However, the spider-leg-like pattern was strongly modulated and obscured by the dominant ATI peaks. In contrast, in the case of a near-single-cycle pulse (Fig. 1d, a cosine-like pulse), we observe that the inter-cycle interference is nearly eliminated, and symmetry has been broken along the ${P}_{z}$ direction when compared to that in the case of a multicycle laser. Figure 1f depicts possible electron trajectories ionized at each half cycle for a 3.3-fs Gaussian pulse. The number of cycles is reduced in this case, and inter-cycle interference is suppressed significantly. Therefore, it allows us to clearly observe intra-cycle interferences, namely a spider-leg-like PH pattern in the ${P}_{z} \,>\, 0$ region and a fishbone-like PH pattern in the ${P}_{z} \,<\, 0$ region. The patterns are indicated by white dashed lines and curves to direct the eyes in Fig. 1d, e. To the best of our knowledge, this is the first direct and simultaneous measurement of fishbone- and spider-leg-like PH patterns in a single-measurement setup. The origin of this specific PH pattern is illustrated in Fig. 1g, h. A spider-leg-like PH pattern is produced by electrons born within the same quarter-cycle (see Fig. 1g, where the green area indicates a quarter-cycle (QC)). These electrons are initially ionized in the negative ${P}_{z}$ direction and then redirected by the laser field when the electric field changes its sign. One of the electrons directly reaches the detector without scattering (direct electrons), while the other is forward-scattered toward the detector. These two electrons coherently interfere at the detector, resulting in a spider-leg-like PH pattern. On the contrary, a fishbone-like PH pattern is generated by electrons born in the first and third QCs (see Fig. 1h). In this scenario, electrons ionized in the first quarter-cycle are backward-scattered and coherently interfere with the direct electrons born in the third QC. It has been well known that backward-scattered electrons are able to approach the ionic core more closely. This characteristic makes them sensitive to the structure of a target, thereby enabling dynamic imaging^9^. The extraction of the target structure utilizing the observed fishbone-like PH pattern will be discussed in Discussion Section. The results of the TDSE simulation for the corresponding experimental conditions are shown in Fig. 1b, e. The simulation parameters were identical to those used in the experiments. We acknowledge a minor difference between the experimental findings and the simulations, recognizing that such distinctions could stem from the utilization of the two-dimensional TDSE method and the detector sensitivity for fine details. However, the experimental results were qualitatively well reproduced by the focal-volume averaged TDSE simulation.

### Control of subcycle electron dynamics

**Fig. 2:**
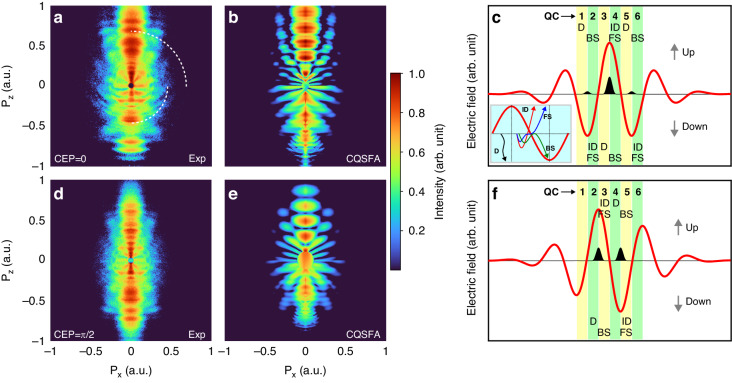
Control of subcycle PH patterns by CEP. **a**, **d** We present the measured PH patterns obtained for cosine-like and sine-like pulses, respectively. **b**, **e** We present the Coulomb quantum-orbit strong-field approximation (CQSFA) calculations for the respective pulses. The simple man model was used to illustrate the direct and rescattered electron trajectories in (**c**) for the cosine-like pulse and in (**f**) for the sine-like pulse, with the inset depicting the electron trajectories. The red solid line represents the electric field shape, whereas the black-filled area shows the estimated ionization rate according to the Ammosov–Delone–Krainov (ADK) model. The yellow and green shaded areas represent every quarter-cycle ionization time window for forward-scattered (FS), backward-scattered (BS), indirect (ID), and direct (D) electrons, with Arabic numbers indicating the origin of the ionization window in terms of a quarter-cycle (QC)

In the previous section, we discussed the observation of spider-leg- and fishbone-like PH patterns at the same time. From the simplified scheme depicted in Fig. 1g, h, it is apparent that both PH patterns originate from electron trajectories that are born within a single optical cycle of the laser field. Here, we demonstrate the control of subcycle electron dynamics using the CEP of the laser field. Figure 2 shows the PH patterns for different CEPs of a near-single-cycle pulse. The figures in the first and second rows are for the cases of cosine-like (${CEP}=0$) and sine-like (${CEP}=\pi /2$) pulses, respectively. The asymmetry in the PH distribution along the ${P}_{z}$ direction in Fig. 2a is more pronounced when compared with Fig. 2d.

In the following, we discuss the underlying physics of these different interference patterns. The TDSE simulation is an ab initio method that can reproduce experimental findings qualitatively. However, it encounters limitations in fully elucidating the intricate underlying physics. In order to understand the experimental observation further and provide the origins of the interference pattern, we used a model-based approach, i.e., the CQSFA method^11,30–32^ (see Supplementary information), to simulate PH patterns for different CEPs of near-single-cycle laser pulses. When compared to the Simple Man Model (SMM)^33,34^-based calculation, the CQSFA theory takes into account the influence of the ionic Coulomb potential on the ionized electrons and thus can better reproduce the observed interference patterns. The CQSFA simulation results for the respective pulses are shown in Fig. 2b, e, which also show the separation of the spider-leg- and fishbone-like PH patterns for the cosine-like laser pulse and the less obvious asymmetric PH patterns for the sine-like laser pulse that are qualitatively consistent with experimental results.

The simultaneous observation of separated spider-leg- and fishbone-like PH patterns can be intuitively understood with the help of the SMM. In general, four types of photoelectron trajectories matter (inset in Fig. 2c): (i) photoelectrons that leave the parent ion and are en route to the detector directly, called direct trajectory (D, black), (ii) indirect trajectory (ID, red), whereas some photoelectrons are returned to the parent ion and could be (iii) forward-scattered (FS, blue), and (iv) backward-scattered (BS, green). The rate of tunnel ionization according to the ADK theory^35^ is also shown in Fig. 2c, f as black bells. As shown in Fig. 2c, in the case of a cosine-like laser pulse, there is only one major ionization event during the QC3–4, at which most electrons are tunnel-ionized, producing both forward- (up direction) and backward- (down direction) scattered electrons, as well as direct electrons. The forward-scattered electron trajectories produce a spider-leg-like pattern in the ${P}_{z} \,>\, 0$ region when they coherently interfere with the indirect electrons born in QC4. Other backward-scattered electron trajectories (born in QC2 and QC6, upward direction) could also contribute but were not significant. Similarly, the backward-scattered electrons born in QC4 result in a fishbone-like PH pattern in the ${P}_{z} \,<\, 0$ region. Other forward-scattered electron trajectories (QC2 and QC6 in the downward direction) had small contributions. Therefore, separated fishbone- and spider-leg-like PH patterns were observed simultaneously. On the other hand, in the case of a sine-like laser pulse (${CEP}=\pi /2$), there are two major tunnel ionization events that occur at two different times with equal probabilities of ionization (Fig. 2f, black bells). In each case, the laser field direction is opposite, so a pair of forward- and backward-scattered trajectories are available in both directions. Accordingly, both fishbone- and spider-leg-like patterns are possible and have been observed. However, the backward-scattered electrons from QC5 cannot obtain much energy from the laser field because they are ionized close to the end of the laser pulse. Thus, only a spider-leg-like PH pattern can be clearly observed in the ${P}_{z} \,>\, 0$ region, in good agreement with the measurement (Fig. 2d). Considering that both spider-leg- and fishbone-like PH patterns are produced by the electron trajectories born within a single optical cycle, it clearly demonstrates that the observed PH patterns are controlled in the subcycle limit by the CEP of the laser pulses.

It has been extensively discussed that spider-leg and fishbone-like PH patterns are useful in enhancing our understanding of electron dynamics^11,36^. Notably, they have been employed to investigate phenomena such as the Gouy phase anomaly in electron scattering^37^ and target structure characterization^9,22^. Until now, the extraction of such information has been impeded by the lack of precise observation of spider-leg- and fishbone-like PH patterns. In the following subsections, we will examine the observed PH patterns to uncover the Gouy phase anomaly and facilitate the extraction of target structures.

### The Gouy phase effect on SFPH patterns

The phase plays an essential role in all kinds of interferometry. In SFPH, as matter-wave interferometry, the phase determines the positions of the fringes (maxima and minima) of the hologram and hence affects the extraction of accurate information about the molecular structure. Thus, all relevant phases related to the direct and scattered electron trajectories should be properly considered. For the scattered electron, its electron wave packet is focused by Coulomb attraction, just as a light wave is focused by a lens, thereby experiencing Gouy’s phase shift. Recently, Brennecke et al.^37^ showed that forward-scattered electron trajectories could acquire the Gouy phase, resulting in shifted interference fringes. However, because of the distorted PH pattern caused by the inter-cycle interference effect, experimental verification of the Gouy phase effect is lacking. In the case of multicycle lasers, an additional time-filtering algorithm is required to remove inter-cycle interference^24^. Therefore, a PH pattern without an inter-cycle interference effect is preferred.

**Fig. 3:**
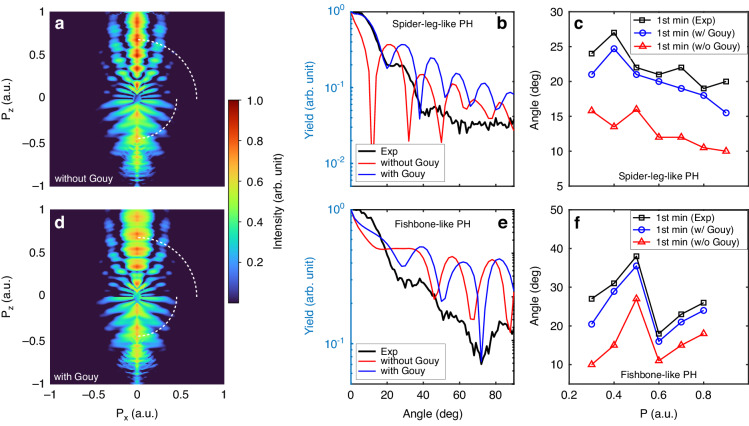
The Gouy phase effect in electron wave. The CQSFA-calculated PH patterns for near-single-cycle pulses (**a**) without and (**d**) with the Gouy phase effect. The lineout extracted from the experimental results for a given momentum P (white dashed arcs in Fig. 2a) was compared with the calculated lineout results along the white dashed arcs and is shown in (**b**) for the spider-leg-like PH pattern and (**e**) for the fishbone-like PH pattern for the near-single-cycle laser. **c**, **f** The experimental first-minimum positions obtained at various momenta are compared with the corresponding results extracted from the CQSFA-calculated results for spider-leg- and fishbone-like PH patterns, respectively

Here, we show that observing an inter-cycle interference-free spider-leg-like PH pattern is useful for directly validating the Coulomb field-induced Gouy phase effect on the electron waves. Figure 3a shows the CQSFA-calculated PHs without the Gouy phase. The simulation parameters are identical to those used in the experiments. A close examination of the experimental (Fig. 2a) and simulated PH reveals that the spider-leg-like pattern in the measurement has a larger width along ${P}_{x}$. To understand this difference, we included Gouy’s phase shift of $-\pi /2$ in the CQSFA simulations for forward-scattered electrons (for details, see Section S2 of Supplementary information). The simulations with the Gouy phase effect (Fig. 3d) shows that the spider-leg-like PH patterns become wider along the ${P}_{x}$ direction, which is now in better agreement with the measurement. For a more quantitative comparison, we plot a cross-sectional profile along the white dashed arc in ${P}_{z} \,>\, 0$ region, integrated over the momentum, $P=0.6{-}0.65$ a.u. range (see Supplementary information for other region of integration, i.e., $P=0.8{-}0.85$ a.u. Section S5), as shown in Fig. 3b. The positions of the interference minima and maxima in the simulation with the Gouy phase effect (blue curve) are well matched with the measurements (black curve); however, those without the Gouy phase effect (red curve) are not. The faint modulation observed above $50$ degree in the simulation was not captured in the experiment due to the limited sensitivity of the detector.

Furthermore, we show that the Gouy phase effect can also be identified in the fishbone-like PH pattern. Similar to the forward-scattered trajectory, the backward-scattered electron trajectories also acquire an additional Gouy phase from the Coulomb focus, leading to a shift of the fringes in the fishbone-like PH pattern. However, the fishbone-like PH pattern is usually difficult to observe directly, and thus, the influence of the Gouy phase on the backward-scattered electrons has received less attention. However, in the present work, we were able to clearly observe the fishbone-like PH pattern using a near-single-cycle laser pulse, presenting the corresponding cross-sectional profile (Fig. 3e) along the white dashed arc in the ${P}_{z} \,<\, 0$ region, integrated over the region of $P=0.4{-}0.45$ a.u (see Supplementary information for other region of integration, i.e., $P=0.6{-}0.65$ a.u., Section S5) in comparison with the experiment. The comparison reveals that the positions of the interference minima and maxima in the simulation with the Gouy phase (blue curve) are in good agreement with the measurements (black curve); however, those without the Gouy phase effect are not. Therefore, we found that both forward- and backward-scattered electrons are impacted by Coulomb focusing.

For the quantitative analysis of the Gouy phase effect, we extracted the first-minimum positions for different momenta from 0.3 to 0.9, with an increment of 0.1 a.u. The signals at the second and third minimum positions in the experimental hologram are rather noisy due to the weakness of the signals themselves and inherent limitations in detector sensitivity. Consequently, the analysis of these minimum positions for robust comparison purposes was not suitable. In Fig. 3c, f, the extracted first-minimum positions are shown along with the corresponding simulation results. The average of the angular position differences of the first-minimum positions between the experiments and simulations are 9.3 and 11.1 degrees for the spider-leg- and fishbone-like PH patterns, respectively, when the Gouy phase effect is not taken into account. This corresponds to ~50% deviation on average between the experiment and calculation. On the other hand, when the Gouy phase effect is considered, the average of the angular position differences reduces significantly to 2.2 and 2.8 degrees for spider-leg- and fishbone-like PH patterns, respectively. This corresponds to ~10% deviation on average between the experiment and calculation. We note the improvement by a factor of 5 when considering the Gouy phase. Hence, an accurate analysis of PH requires consideration of the Gouy phase effect.

## Discussion

### The dependence of the fishbone-like PH pattern on the internuclear separation

It is well known that the fishbone-like PH pattern results from the backward-scattered electron trajectory, which can get close to the ionic core during rescattering^9,38^. Thus, the fishbone-like PH pattern is able to provide information regarding the target structure. For example, backscattering holograms have been used to probe molecular dynamics in small molecules^22^ and the fishbone-like PH pattern was obtained by the differential holography method, where the difference in photoelectron momentum distribution between the aligned and anti-aligned molecules was utilized to extract the fishbone-like PH pattern. In the current study, we directly measured a clear fishbone-like PH pattern in a single-measurement setup. In comparison with the theoretical results from the CQSFA method, we demonstrate that the measured fishbone-like PH pattern can indeed be used to determine the internuclear separation of a target molecule.

**Fig. 4:**
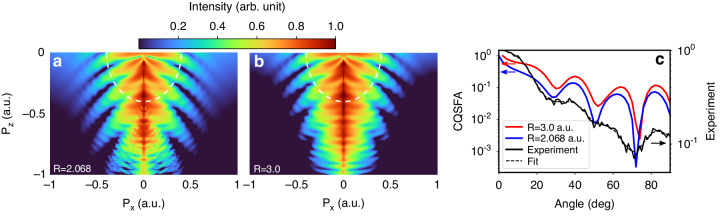
Fishbone-like PH pattern and interatomic distance. The CQSFA simulation for the PH pattern in the ${P}_{z} \,<\, 0$ region with the internuclear separation of (**a**) *R* = 2.068 a.u. and (**b)** *R* = 3.0 a.u. **c** The extracted fringe position (or angular dependence) of the fishbone-like PH pattern with a momentum of *P* = 0.4 a.u. (along the white dashed arc in (**a**, **b**)) in comparison with the experimental data is shown

In Fig. 4a, b, the simulated PH patterns in the ${P}_{z} \,<\, 0$ region using the CQSFA method are shown for different internuclear separations with $R=2.068$ a.u. and $R=3.0$ a.u., respectively. The main features of the fishbone-like PH are the same when the value of $R$ is increased; however, closer examination reveals that the positions of the interference fringes change as $R$ changes. To examine the change in the interference pattern more clearly, the cross-sectional profiles of the fishbone-like PH pattern at $P=0.4$ a.u. (i.e., following the white dashed arcs in Fig. 4a, b) are plotted in Fig. 4c. With an increase in $R$, the dips in the curve shift to the higher angle region. Therefore, one can extract molecular structural information by fitting the positions of these interference minima. Comparing the simulation results for different *R* with the experimental data, we obtain the range of *R* in which the three interference minima lie in the experimental error range (see details in Supplementary information). Our result shows that the fitted internuclear distance *R* falls between 1.96 a.u. and 2.2 a.u., with an average of $R=2.08\pm 0.12$ a.u., which agrees well with the atomic separation in the literature^39–42^: $R=2.068$ a.u. Hence, the fishbone-like PH pattern produced by a near-single-cycle pulse carries out the correction information of the molecular structure, indicating that it can be used for the dynamical imaging of molecular structures.

### Extension to other molecules

**Fig. 5:**
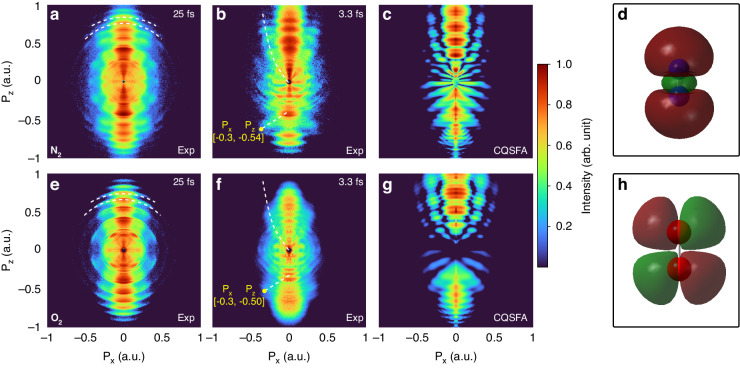
Comparison of experimental results for N_2_ and O_2_ molecules. **a**–**d** represent N_2_, while **e**–**h** correspond to O_2_. Results obtained under multicycle laser pulses are shown in (**a**, **e**), while near-single-cycle laser experiments are presented in (**b**, **f**). The corresponding CQSFA simulations are shown in (**c**, **g**). The HOMO structures for each molecule are depicted in (**d**, **h**), providing insights into the observed variations in experimental data

In order to validate and expand the approach, we also conducted additional experiments using O_2_ molecules, which possess different highest-occupied molecular orbitals (HOMO) from those of N_2_. The experimental results are shown in Fig. 5 (second row) in comparison with N_2_ molecules (first row), along with their HOMO structures. As in the case of N_2_, the characteristic ATI-peak structure is prominent and obscures the spider-leg-like PH pattern in the multicycle case (see Fig. 5e). It is noteworthy that the distinction between two molecules is noted in the low-energy region (<0.35 a.u.). This is ascribed to the difference in HOMO structure of each molecule (see Fig. 5d, h). Furthermore, when O_2_ molecules are ionized using CEP-stabilized near-single-cycle pulses, the ATI-peak structure is significantly suppressed, and an asymmetric PH pattern emerges (see Fig. 5f), exhibiting similar attributes to N_2_: a spider-leg-like PH pattern in the ${P}_{z} \,>\, 0$ region and a fishbone-like PH pattern in the ${P}_{z} \,<\, 0$ region. This experimental finding highlights the versatility of the near-single-cycle SFPH approach, showcasing its applicability across different molecular entities. However, a closer inspection reveals that the position of the fishbone PH in O_2_ is shifted to the lower energy region. Such an energy shift of the fishbone PH can also be observed in the CQSFA simulation of O_2_, indicating that the fishbone-like PH is sensitive to the structure of the molecule target (*R* = 2.068 a.u. for N_2_, $R=2.281$ a.u. for O_2_)^43^. In addition, it is noteworthy that the fishbone-like PH pattern in O_2_ appears less pronounced when compared to its counterpart in N_2_. The main reason is that the HOMO of the O_2_ molecule consists only of $p$-state atoms, and thus the corresponding two-center interference term has only a term: 2i sin*(****p****∙****R***⁄2*)*. As a result, for smaller momenta ${\boldsymbol{p}}$, the photoelectron amplitude will decrease accordingly.

In conclusion, a CEP-stabilized near-single-cycle laser pulse significantly suppressed the inter-cycle interference effects (ATI peaks) in the SFPH experiment, allowing the emergence and control of subcycle-relevant PH patterns. To the best of our knowledge, this is the first direct experimental observation of a fishbone-like PH pattern, with a clear distinction and separation from spider-leg-like PH patterns. On the other hand, using the CEP of a laser, we also demonstrated that the electron dynamics in the rescattering process can be controlled on an attosecond timescale. When the cosine-like pulse is used, the spider-leg-like (${P}_{z} \,>\, 0$) and fishbone-like (${P}_{z} \,<\, 0$) PH patterns could coexist in a single photoelectron momentum distribution. The experimental results are qualitatively reproduced using the TDSE and CQSFA methods. Our analysis using the CQSFA method revealed the significant influence of the Coulomb field-induced Gouy phase effect on the observed PH patterns. This effect plays a crucial role in determining the fringe extrema and must be taken into account for a precise analysis of electron dynamics. The fishbone-like PH pattern observed in the experiment was utilized to deduce the structure of the target molecule by comparing it with theoretical simulations. These findings not only deepen our understanding of electron scattering near the ionic core but also present a promising avenue for the development of a single-cycle-based SFPH method for dynamic molecular imaging and quantum control.

Unlike the methods presented in literatures, our approach distinguishes itself by its simplicity and the ability to be executed with a single-measurement setup. Conventional measurement techniques such as differential holography^22^ and the two-color method^18,44^, require at least two measurements or intricate experimental setups to achieve comparable results. Our approach not only reduces the number of required measurements but also mitigates the complexities associated with experimental arrangements. Moreover, the adoption of a single-measurement setup enhances the versatility of the method. For instance, by integrating a secondary beam, one could enhance its adaptability, creating a powerful technique. This additional beam could function as precise control over electron ionization timing. Moreover, the inclusion of a molecular alignment beam opens avenues for exploring orientation-dependent backscattering and site selectivity, particularly for non-homonuclear and linear molecules. Furthermore, our method holds potential for studying atomic targets, and extending its reach to encompass more intricate (i.e., polar or non-homonuclear) molecules. These future directions can pave the way for refining our method and present exciting prospects for further development in this field.

## Materials and methods

Details of theoretical and experimental methods can be found in the Supplementary Information.

### Supplementary information

SI_final
