# Supplementary material for: Strong-field photoelectron holography in the subcycle limit
Source: Light Sci Appl. 2024 May 8;13:108. doi: 10.1038/s41377-024-01457-7 (PMC11076600; doi:10.1038/s41377-024-01457-7)
Supplement: Supplementary file 1 — SI_final [file 41377_2024_1457_MOESM1_ESM.pdf]

Supplementary Information for
Strong-field photoelectron holography in the subcycle limit

Tsendsuren Khurelbaatar[1,2], Jaewuk Heo[1,2], ShaoGang Yu[3], XuanYang Lai[3,4*], XiaoJun Liu[3], and Dong Eon Kim[1,2*]

[1]Center for Attosecond Science and Technology, Department of Physics, Pohang University of Science and Technology, Pohang, Gyeongbuk 37673, Korea.
[2]Max Planck POSTECH/KOREA Research Initiative, Pohang, Gyeongbuk 37673, Korea
[3]State Key Laboratory of Magnetic Resonance and Atomic and Molecular Physics, Wuhan Institute of Physics and Mathematics, Innovation Academy for Precision Measurement Science and Technology, Chinese Academy of Sciences, Wuhan 430071, China
[4]Wuhan Institute of Quantum Technology, Wuhan, 430206, China

E-mails: xylai@wipm.ac.cn, kimd@postech.ac.kr

S1. Time-dependent Schrödinger equation (TDSE)

In this work, we numerically solve the two-dimensional TDSE in the length gauge using the single-active electron approximation (in atomic units):

$$i\frac{\partial}{\partial t}\psi(\boldsymbol{r},t) = \left[\frac{\boldsymbol{p}^2}{2} + V(\boldsymbol{r}) - \mathbf{E}(t)\cdot\boldsymbol{r}\right]\psi(\boldsymbol{r},t) \qquad \text{(S1)}$$

where $\psi(\boldsymbol{r},t)$ denotes the time-dependent electron wavefunction, $V(\boldsymbol{r})$ is the Coulomb potential of a target system, and $E(\boldsymbol{t})$ is the electric field of the laser pulse. For the N_2 molecule, the detailed formula of the Coulomb potential can be found in Refs[1,2].

The electric field of a near-single-cycle linearly polarized laser pulse is given by $E(t) = E_0\cos(\omega t/2N_c)^2\cos(\omega t+\phi)$, where E_0 is the peak electric field, ω is the photon energy, N_c denotes the number of optical cycles, and ϕ is the carrier-envelope-phase. In our calculation, the initial state of the bound electron was obtained using an imaginary-time propagation method, and the propagation of the electron wavefunction in the laser electric field was implemented using a split-operator method[3]. It is usually time-consuming to solve the Schrödinger equation. In the practical calculation, a splitting algorithm[4,5] is used, in which the

electron wavefunction is split into the inner spatial region ($\boldsymbol{r} \in (\mathbf{0}, \boldsymbol{r_c})$) and outer spatial regions ($\boldsymbol{r} > \boldsymbol{r_c}$): $\psi(\boldsymbol{r}, t) = \psi_{in}(\boldsymbol{r}, t) + \psi_{out}(\boldsymbol{r}, t)$. The wavefunction in the inner region is numerically propagated under the full Hamiltonian, while in the outer region, the electron-core interaction is much smaller than the electron-laser interaction; hence, the Coulomb potential can be neglected. Thus, the wavefunction in the outer region is analytically propagated using the Volkov Hamiltonian: $H_V = \frac{p^2}{2} - \mathbf{E}(t) \cdot \boldsymbol{r}$. At each time step, we first evolve the inner wavefunction, and part of the wave packet will enter the outer region. Then we transform the wavefunction in the outer region ψ_{out} to the momentum space:

$$C(\boldsymbol{p}, t_i) = \frac{1}{2\pi} \int \psi_{out}(t_i) e^{-i[\boldsymbol{p}+\mathbf{A}(t_i)]\cdot \boldsymbol{r}} d\boldsymbol{r} \tag{S2}$$

Thus, the outer wavefunction is propagated from t_i to the end of the pulse as

$$\psi_{out}(\infty, t_i) = \frac{1}{2\pi} \int \bar{C}(\boldsymbol{p}, t_i) d\boldsymbol{p} \tag{S3}$$

where

$$\bar{C}(\boldsymbol{p}, t) = e^{-i \int_t^{\infty} [\boldsymbol{p}+\mathbf{A}(\tau)]^2/2 d\tau} C(\boldsymbol{p}, t) \tag{S4}$$

After the end of the laser pulse, the final photoelectron momentum distribution is obtained as

$$\frac{dM(\boldsymbol{p})}{dpd\theta} = \left| \sum_t \bar{C}\,(\boldsymbol{p}, t) \right|^2 \tag{S5}$$

where θ is the direction of the final momentum of the photoelectron. To compare with experimental results, the focal volume averaging has also been done. Through the focus, the laser intensity changes. Hence, the photoelectron angular distributions have been calculated for different laser intensities along the propagation direction. These photoelectron distributions have been integrated over the spatial profile of a Gaussian laser focus[6,7].

S2. Coulomb quantum-orbit strong-field approximation (CQSFA) theory

To simulate the two kinds of PH patterns, a CQSFA theory is used[8–11]. The CQSFA theory is derived from the exact transition amplitude of an electron from an initial state $|\psi_0\rangle$ of an atom or a molecule to the continuum state $|\psi_{\boldsymbol{p}}\rangle$ with drift momentum $\boldsymbol{p}$

$$M(\boldsymbol{p}) = -i \lim_{t\to\infty} \int_{-\infty}^{t} dt' \langle \psi_{\boldsymbol{p}} | U(t,t') H_I(t') | \psi_0(t') \rangle \tag{S6}$$

where $H_I(t') = -\boldsymbol{r}\cdot\mathbf{E}(t')$ is the electron-core interaction and $U(t,t')$ is the time-evolution operator. In our work, the electron wavefunction $|\psi_0\rangle$ is written as a weighted sum of the constituent atomic orbitals of each core, according to the theory of the linear combination of atomic orbitals:

$$\psi_0(r) = \sum_{a=2s,2p} |c_a| e^{i\theta_a} [\psi_a(r+R/2) + S_a \psi_a(r-R/2)] \tag{S7}$$

where $\psi_a(r+R/2)$ and $\psi_a(r-R/2)$ denote bound states of one nitrogen atom and another nitrogen atom, respectively and $|c_a|$ is the weighted coefficients with the phase of θ_a. By introducing the closure relation $\int d\tilde{p}_0 |\tilde{p}_0\rangle\langle\tilde{p}_0| = 1$, Eq. (S6) is written as

$$M(\boldsymbol{p}) = -i \lim_{t\to\infty} \int_{-\infty}^{t} dt' \int d\tilde{\boldsymbol{p}}_0 \langle \psi_{\mathbf{p}} | U(t,t') | \tilde{\boldsymbol{p}}_0 \rangle \langle \tilde{\boldsymbol{p}}_0 | H_I(t') | \psi_0(t') \rangle \tag{S8}$$

where $|\tilde{\boldsymbol{p}}_0\rangle = \boldsymbol{p}_0 + \boldsymbol{A}(t')$ denotes the velocity of the electron at the initial time. Note that the bound states of the system have been neglected in the above-stated closure relation. Physically, this approximation means that we are excluding the processes involving the excited bound states at the instant t' and focusing on the bound-continuum transitions. Furthermore, by employing the Feynman path integral formalism and saddle-point approximation, the transition amplitude can be rewritten as:

$$M(\boldsymbol{p}) \propto -i\lim_{t\to\infty}\sum_{s}\left\{\det\left[\frac{\partial \boldsymbol{p}_s(t)}{\partial \boldsymbol{r}_s(t_{0,s})}\right]\right\}^{-1/2} e^{iS(\boldsymbol{p}_s,\boldsymbol{r}_s,t_{0,s},t)}$$

$$\times C(t_{0,s})\sum_{a}\langle \boldsymbol{p}_s(t_{0,s}) + \mathbf{A}(t_{0,s})|\hat{H}_I(t_{0,s})|\psi_a\rangle \tag{S9}$$

$$\times c_a\left(e^{i\boldsymbol{p}\cdot\boldsymbol{R}/2} + s_a e^{-i\boldsymbol{p}\cdot\boldsymbol{R}/2}\right)$$

where the index s denotes different quantum trajectories with the ionization time $t_{0,s}$, $S(\boldsymbol{p}_s, \boldsymbol{r}_s, t_{0,s}, t)$ is the action of the quantum trajectory, and the index a represents the component atomic orbitals $|\psi_a\rangle$ of the electron wavefunction with the expansion coefficients c_a according to the theory of the linear combination of atomic orbitals. The last term $e^{i\boldsymbol{p}\cdot\boldsymbol{R}/2} + s_a e^{-i\boldsymbol{p}\cdot\boldsymbol{R}/2}$ corresponds to the two-center interference from the two cores of N_2 molecule, where the coefficient $s_a = (-1)^{l_a}$ for the molecular orbital with g symmetry, and $s_a = (-1)^{l_a+1}$ for the molecular orbital with u symmetry. The coefficient l_a denotes the orbital quantum number of the atomic orbitals. Because the ionization of the HOMO of N_2 molecule is dominant, the initial state $|\psi_0\rangle$ is assumed to be in the HOMO state in our work. The different quantum trajectories are obtained from three saddle-point equations:

$$\begin{cases} [\boldsymbol{p}_0 + \boldsymbol{A}(t_0)]^2/2 = -I_p, \\ \dot{\boldsymbol{p}}(\tau) = -\nabla_{\boldsymbol{r}} V(\boldsymbol{r}(\tau)), \\ \dot{\boldsymbol{r}}(\tau) = \boldsymbol{p}(\tau) + \boldsymbol{A}(\tau), \end{cases} \tag{S10}$$

which are solved using an iteration scheme for any given final momentum. Once the saddle points are solved, we can proceed to solve Newton's equations to obtain the trajectories. By substituting these trajectories into the derived transition formula (S9), we can determine the corresponding photoelectron amplitude.

In our work, we also take into account the Gouy phase effect on SFPH within the CQSFA method. Specifically, when incorporating the Gouy phase, we manually add a phase shift of $-\pi/2$ to the action of the forward- and backward-scattered trajectories in Eq. (S9).

Conversely, in simulations where the Gouy phase is not considered, we omit this additional phase adjustment. Similar approaches have been discussed in prior literature for the spider PH[12].

S3. Experimental setup

A set of strong-field photoelectron holography experiments was carried out using a linearly polarized carrier-envelope-phase (CEP)-stabilized 3.3-fs near-single-cycle laser at 723 nm and a 25-fs multicycle laser at 800 nm. In both cases, the laser polarization direction was parallel to the detector plane (see Fig. S1(b)). The experimental setup is schematically shown in Fig. S1. It should be noted that the specific wavelengths used in our experiment were determined by the inherent capabilities of our laser source systems. Driving laser wavelength could influence the spacing between above-threshold ionization peaks in the photoelectron momentum distribution. The photon energy difference between the 800 nm (1.55 eV) and 723 nm (1.71 eV) wavelengths, on the other hand, is 0.16 eV (or 0.005 a.u.) and has no significant effect on the experimental observables. For the multicycle experiment, Ti:sapphire amplifier output was picked up and directed to the experimental chamber. The intensity of the multicycle laser pulse is controlled with a half-wave plate (HWP) and a linear polarizer (LP).

For the near-single-cycle experiment, we begin with CEP-stabilized 1.5 mJ, 25 fs near-infrared (NIR) pulses from the output of the Ti:sapphire amplifier. The amplifier output pulse is directed and focused on a Ne gas-filled hollow-core fiber (inner diameter 400 μm, 1 m long) to achieve spectral broadening. The spectrally broadened pulses are then compressed using 7 pairs of chirped mirrors and a fused silica wedge pair (50 × 25 mm, wedge angle 4 degree). One of the wedge pairs was installed in a motorized linear stage for fine control of the wedge position for the CEP and dispersion scan experiments. To achieve a near-single-cycle laser pulse[13,14], we further compress the third-order dispersion (TOD) using a 1.5-mm-thick ammonium dihydrogen phosphate (ADP) crystal and another pair of chirped mirrors. For the

pulse characterization, we implemented a dispersion scan[15] setup, and experimental details will be discussed in Section S4.

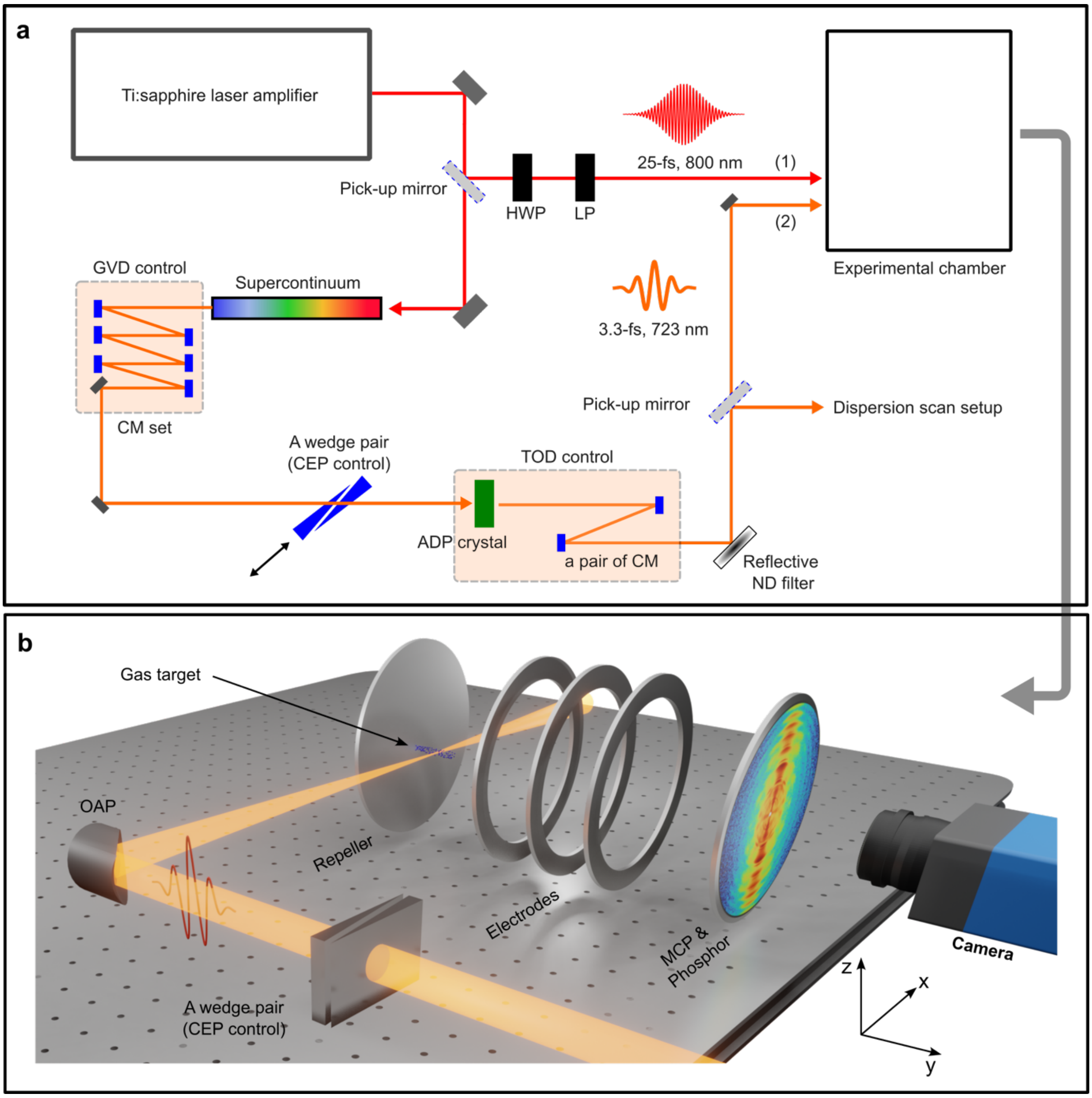

Figure S1. Schematic representation of the experimental setup. Sources for near-single-cycle and multicycle laser pulse generation are shown in **(a)**. **(b)** depicts the VMI setup. The laser polarization direction was parallel to the detector plane. HWP: half-wave plate, LP: linear polarizer, CM: chirped mirror, GVD: group velocity dispersion, TOD: third-order dispersion, ADP: ammonium dihydrogen phosphate, ND: neutral density, OAP: off-axis parabolic mirror, MCP: microchannel plate

In the case of a near-single-cycle pulse, laser intensity was adjusted using a broadband reflective neutral density filter. Inside the experimental chamber, the laser pulses were focused on the gas target using an off-axis parabolic (OAP) mirror with a $f = 150$ mm focal length (see Fig. S1(b)). For the near-single-cycle experiment, estimated laser peak intensities at the interaction point were $\sim 2.5 \times 10^{14}$ W cm^{-2} for N_2 and $\sim 1.7 \times 10^{14}$ W cm^{-2} for O_2. A gas target was injected via an effusive nozzle (50 μm) integrated into the repeller plate of the velocity map imaging (VMI) spectrometer[16]. The resulting photoelectron momentum distributions were focused on a MCP-phosphor screen assembly detector using electrostatic lenses and recorded using a CMOS camera. The energy calibration of the VMI spectrometer was carried out by measuring the above-threshold ionization peaks of Xe gases with multicycle laser pulses. The laser peak intensities were estimated by analyzing the $2U_p$ classical cutoffs from the photoelectron energy distribution, where the U_p is ponderomotive energy.

S4. Dispersion compensation and pulse characterization

For the near-single-cycle pulse characterization, we utilized the dispersion scan method and the schematic layout depicted in Figure S2.

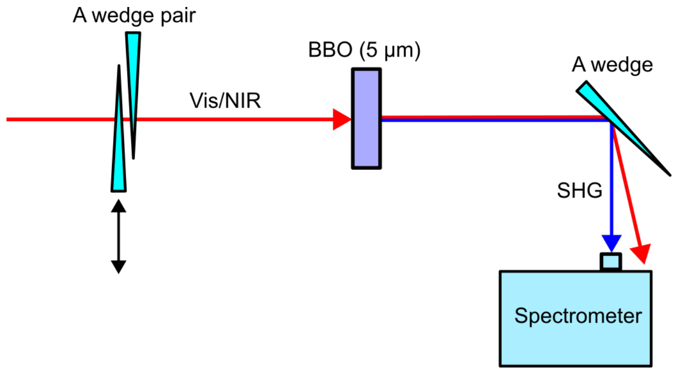

Figure S2. Schematic of dispersion scan setup. Ultrashort pulses are sent to an ultrathin (5 μm) BBO crystal, and the generated SHG signal is separated from the fundamental by wedge reflection at the Brewster angle and measured with a spectrometer while the dispersion is varied with wedge insertion.

After proper dispersion control, the generated near-single-cycle laser pulses are sent to the dispersion scan setup and focused on the beta barium borate (BBO) crystal for second harmonic

generation (SHG). Generated second harmonics and fundamental NIR beams propagated collinearly after the BBO, and the second harmonic signal was separated from its fundamental using a wedge reflection at the Brewster angle and detected by a spectrometer (HR4000, Ocean Optics).

A dispersion scan trace is collected when scanning a wedge with a motorized linear stage with a home-made Labview program. The pulse characteristics are retrieved with the differential evolution algorithm[17], which is displayed in Fig. S3.

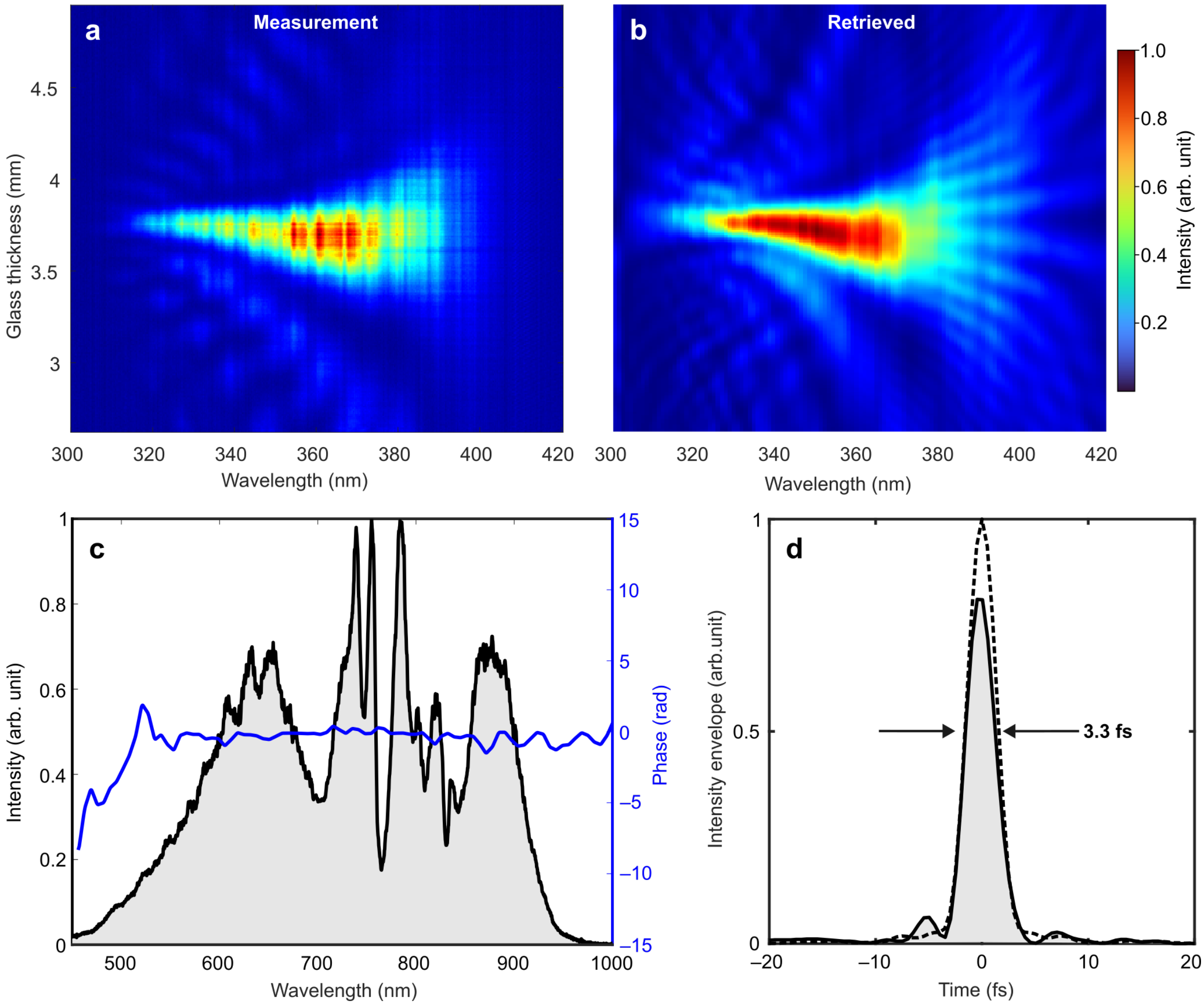

Figure S3. Dispersion scan measurement of a generated near-single-cycle laser pulse. (a) Measured trace **(b)** Retrieved trace. **(c)** The corresponding supercontinuum output spectrum (black) and the reconstructed spectral phase (blue). **(d)** Reconstructed near-single-cycle pulse intensity envelope (solid curve) exhibiting a pulse duration of 3.3 fs. The reconstructed envelope is nearly identical to the Fourier transform-limited envelope (dashed curve), which is 3.2 fs.

S5. Gouy phase extraction at different momentum positions

To further validate the Gouy phase, we extracted Gouy phase at different momentum position,

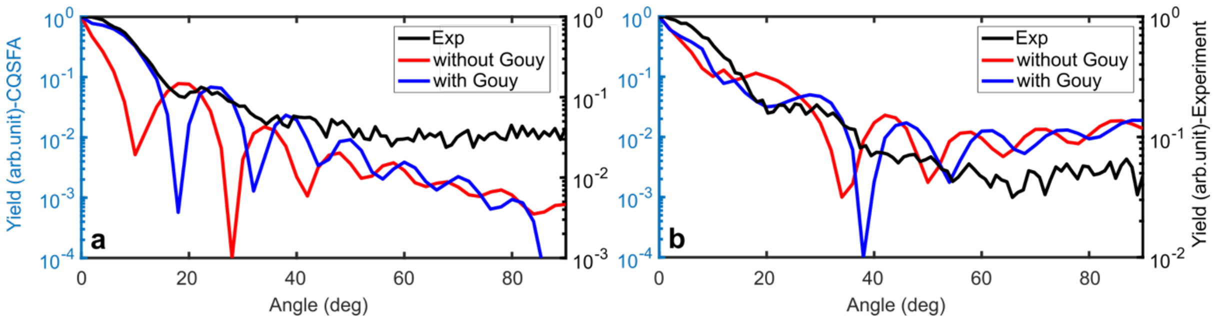

Figure S4. The Gouy phase effect in electron wave. The lineout extracted from the experimental results was compared with the CQSFA-calculated lineout results along the white dashed arcs (see Fig. 3 in the main text). **(a)** For the spider-leg-like PH paIern with the momentum P = 0.8 − 0.85 a.u. and **(b)** for the fishbone-like PH pattern with the momentum P = 0.6 − 0.65 a.u. for the near-single-cycle laser.

S6. Accuracy of internuclear separation determination

To show the degree of accuracy of internuclear separation determination, we have considered the uncertainty of the measurement. In Fig. S5(a), we displayed a collection of data sets obtained from the 2D photoelectron momentum distribution at a momentum position of P = 0.4 a.u. in the $P_z < 0$ direction, along the path indicated by the white dashed arc in Fig. 4(a) of the manuscript.

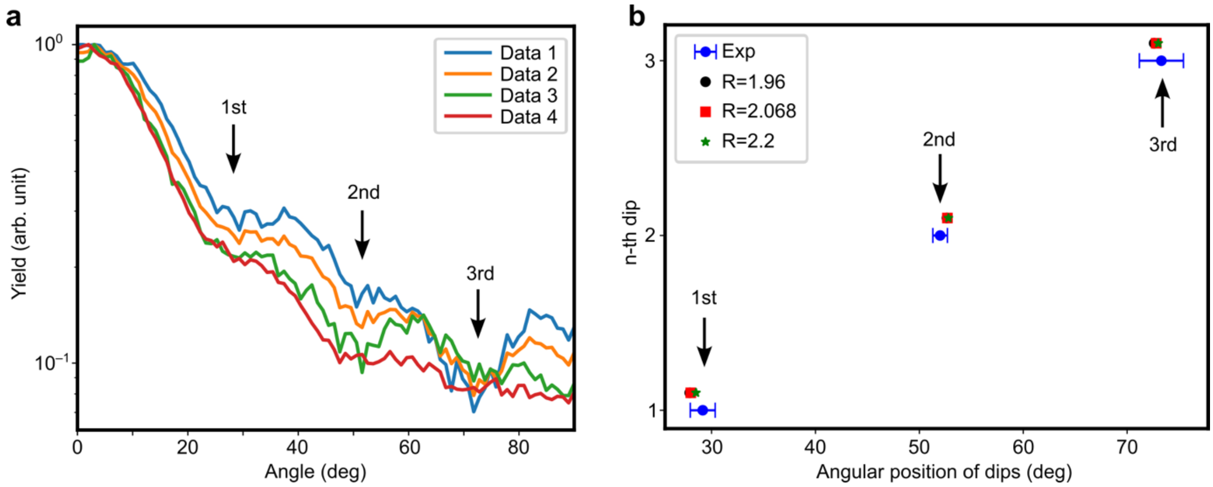

Figure S5. Accuracy of internuclear separation. (a) A collection of data sets obtained from the 2D photoelectron momentum distribution at a momentum position of P = 0.4 a.u. in the $P_z < 0$ direction. **(b)** (Blue dots with error bar) Positions of three minima observed in the measurement (see Fig. 4(c) in the

manuscript), along with their uncertainties. (Black, red, and green) The corresponding positions of these three minima obtained from theoretical simulations with different R values.

It should be noted, however, that these data sets were collected on different dates, and the data integration time was different while the other experimental parameters were similar. Each data set was obtained by averaging 8 images, and the highest signal-to-noise ratio (data 1) was observed with the longest integration time of 8 seconds. As the integration time decreases (8, 6, 4, and 2 seconds, respectively), the signal-to-noise ratio correspondingly gets smaller. Notably, for the smallest integration time (2 seconds), the fringe minimum positions become less distinct; the fringe minimum positions are still discernible and informative. To determine the uncertainties in the internuclear separation, we obtained angular deviations from Fig. S5(a) and plotted them as horizontal error bars in Fig. S5(b). One of the causes of fringe minimum uncertainty is the CEP stability of the laser pulses. Therefore, it's worth noting that good CEP stability is desired for the internuclear separation experiment and the accuracy of the result. Then, we numerically simulate the positions of these three minima according to the CQSFA model. By varying the value of R and comparing the numerical results with the experimental data, we find that the first minimum obtained from theory lies outside the experimental error range when $R < 1.96$ a.u., while the second minimum obtained from theory lies outside the error range when $R > 2.2$ a.u (see Fig. S5(b)). Therefore, the estimated internuclear distance R falls between 1.96 a.u. and 2.2 a.u., and we approximately express the corresponding fitted distance as $R = 2.08 \pm 0.12$ a.u.

References

1. Yu, S. et al. Photoelectron holography from multiple-return backscattering electron orbits. *Physical Review A* **101**, 023414 (2020).
2. Peters, M. et al. Laser-induced electron diffraction: A tool for molecular orbital imaging. *Physical Review A* **85**, 053417 (2012).
3. Feit, M. D. et al. Solution of the Schrödinger equation by a spectral method. *Journal of Computational Physics* **47**, 412–433 (1982).
4. Chelkowski, S. et al. Electron-nuclear dynamics of multiphoton H_2^+ dissociative ionization in intense laser fields. *Physical Review A* **57**, 1176–1185 (1998).
5. Tong, X. M. et al. Numerical Observation of the Rescattering Wave Packet in Laser-Atom Interactions. *Physical Review Letter* **99**, 093001 (2007).
6. Kopold, R. et al. Channel-closing effects in high-order above-threshold ionization and high-order harmonic generation. *Journal of Physics B: Atomic, Molecular and Optical Physics* **35**, 217 (2002).
7. Hasović, E. et al. Simulation of above-threshold ionization experiments using the strong-field approximation. *Laser Physics* **17**, 376–389 (2007).
8. Faria, C. F. de M. et al. It is all about phases: ultrafast holographic photoelectron imaging. *Reports on Progress in Physics* **83**, 034401 (2020).
9. Maxwell, A. S. et al. Coulomb-corrected quantum interference in above-threshold ionization: Working towards multitrajectory electron holography. *Physical Review A* **96**, 023420 (2017).
10. Lai, X.-Y. et al. Influence of the Coulomb potential on above-threshold ionization: A quantum-orbit analysis beyond the strong-field approximation. *Physical Review A* **92**, 043407 (2015).

11. Lai, X.-Y. et al. Reconstructing Molecular Orbitals with Laser-Induced Electron Tunneling Spectroscopy. *Ultrafast Science* **4**, 0038 (2024).

12. Brennecke, S. et al. Gouy's Phase Anomaly in Electron Waves Produced by Strong-Field Ionization. *Physical Review Letter* **124**, 153202 (2020).

13. Timmers, H. et al. Generating high-contrast, near single-cycle waveforms with third-order dispersion compensation. *Optics Letter* **42**, 811–814 (2017).

14. Seo, M. et al. High-contrast, intense single-cycle pulses from an all thin-solid-plate setup. *Optics Letter* **45**, 367–370 (2020).

15. Silva, F. et al. Simultaneous compression, characterization and phase stabilization of GW-level 1.4 cycle VIS-NIR femtosecond pulses using a single dispersion-scan setup. *Optics Express* **22**, 10181–10191 (2014).

16. Kling, N. G. et al. Thick-lens velocity-map imaging spectrometer with high resolution for high-energy charged particles. *Journal of Instrumentation* **9**, P05005 (2014).

17. Escoto, E. et al. Advanced phase retrieval for dispersion scan: a comparative study. *Journal of the Optical Society of America B* **35**, 8–19 (2018).
